# Supplementary material for: Identification of key genes in bovine muscle development by co-expression analysis
Source: PeerJ. 2023 Apr 12;11:e15093. doi: 10.7717/peerj.15093 (PMC10105563; doi:10.7717/peerj.15093)
Supplement: Supplemental Information 11 [file peerj-11-15093-s011.docx]

**Supplementary Table 11： Nucleotide sequences of the primers used for Real-time fluorescence quantitative PCR**

| Genes | Direction | Primer sequence (5’-3’) | Amplification size |
| --- | --- | --- | --- |
| MyHC | F | CTGGAATCCGGAGGCAGAA | 105 bp |
|  | R | TTTTCGAAGGTAGGGAGCGG |  |
| MyoG | F | GGCTGACAAATGCCAGACTATCC | 140 bp |
|  | R | TGGTCCCTTGCTTTATCTCCCT |  |
| Atp2a1 | F | ACCATTGGCTCATGGTCCTC | 108 bp |
|  | R | AGGAGGGGGAAACGGGTTAT |  |
| Tmod4 | F | GCGTGAGAATCGTAGCCTCCAG | 81 bp |
|  | R | GCCTTCAGCACAGCCATAAGC |  |
| Lmod3 | F | CCAACAGGGAACTTCGACCA | 86 bp |
|  | R | ACACGTTCGTCTTCCAGCAT |  |
| Mybpc2 | F | GCGAGTATGAGAGGATCGCC | 91 bp |
|  | R | TTCTTGACCTCGACCTTGGC |  |
| Ryr1 | F | GATGTACAGTCAGGTGGCTCG | 131 bp |
|  | R | GCACACATGTTCAGGCCGAT |  |
| GAPDH | F | ACAGTCAAGGCAGAGAACGG | 98 bp |
|  | R | CCAGCATCACCCCACTTGAT |  |
