# Supplementary material for: Identification of key genes in bovine muscle development by co-expression analysis
Source: PeerJ. 2023 Apr 12;11:e15093. doi: 10.7717/peerj.15093 (PMC10105563; doi:10.7717/peerj.15093)
Supplement: Supplemental Information 12 [file peerj-11-15093-s012.docx]

**Supplementary Table 12：Real-time fluorescence quantitative reaction program**

qRT-PCR reaction program

| Procedure | Temperature | Time | Cycles |
| --- | --- | --- | --- |
| Preincubation | 95℃ | 600 s | 1 |
|  | 95℃ | 10 s |  |
| 3 Step Amplification | 63℃ | 20 s | 35 |
|  | 72℃ | 15 s |  |
|  | 95℃ | 10 s |  |
| Melting | 65℃ | 60 s | 1 |
|  | 97℃ | 1 s |  |
| Cooling | 97℃ | 30 s | 1 |
